# Current Insights and Novel Cardiovascular Magnetic Resonance-Based Techniques in the Prognosis of Non-Ischemic Dilated Cardiomyopathy

**DOI:** 10.3390/jcm13041017

**Published:** 2024-02-09

**Authors:** Francesco Perone, Ilaria Dentamaro, Lucia La Mura, Angeliki Alifragki, Maria Marketou, Elena Cavarretta, Michael Papadakis, Emmanuel Androulakis

**Affiliations:** 1 Cardiac Rehabilitation Unit, Rehabilitation Clinic “Villa delle Magnolie”, 81020 Castel Morrone, Italy; francescoperone1988@gmail.com; 2 Cardiology Department, Hospital of Policlinico of Bari, 70124 Bari, Italy; ilaria.dentamaro@gmail.com; 3 Department of Advanced Biomedical Sciences, University Federico II of Naples, 80133 Naples, Italy; lucia.lamura@hotmail.it; 4 Department of Cardiology, University General Hospital of Heraklion, 71500 Crete, Greece; alifrangie@gmail.com (A.A.); maryemarke@yahoo.gr (M.M.); 5 Department of Medical-Surgical Sciences and Biotechnologies, Sapienza University of Rome, Corso Della Repubblica, 79, 04100 Latina, Italy; elena.cavarretta@uniroma1.it; 6 Mediterranea Cardiocentro, 80122 Napoli, Italy; 7 Department of Cardiology, St George’s University, London SW170QT, UK; mipapada@sgul.ac.uk; 8 Cardiovascular Imaging Centre, Royal Brompton Hospital, Guy’s and St Thomas NHS Foundation Trust, London SW3 6NP, UK

**Keywords:** cardiac magnetic resonance, dilated cardiomyopathy, myocardial fibrosis, parametric mapping, extracellular volume, gray zone fibrosis

## Abstract

Cardiac magnetic resonance (CMR) imaging has an important emerging role in the evaluation and management of patients with cardiomyopathies, especially in patients with dilated cardiomyopathy (DCM). It allows a non-invasive characterization of myocardial tissue, thus assisting early diagnosis and precise phenotyping of the different cardiomyopathies, which is an essential step for early and individualized treatment of patients. Using imaging techniques such as late gadolinium enhancement (LGE), standard and advanced quantification as well as quantitative mapping parameters, CMR-based tissue characterization is useful in the differential diagnosis of DCM and risk stratification. The purpose of this article is to review the utility of CMR in the diagnosis and management of idiopathic DCM, as well as risk prediction and prognosis based on standard and emerging CMR contrast and non-contrast techniques. This is consistent with current evidence and guidance moving beyond traditional prognostic markers such as ejection fraction.

## 1. Introduction

Dilated cardiomyopathy (DCM) is the most common cardiomyopathy originating from multiple causes which can be clinically presented with heart failure, occasionally requiring heart transplantation, and an increased risk of ventricular arrhythmias and/or sudden cardiac death [1]. Idiopathic DCM is characterized by a dilated left ventricle (LV) with impaired systolic function in the absence of abnormal loading conditions (e.g., uncontrolled hypertension, valvular heart disease, congenital heart disease) or significant coronary artery disease [2].

The prevalence of DCM ranges from 1/2500 up to 1/250 people, mainly due to changes in diagnostic criteria and geographical variations [3]. Recent studies using genetic screening have suggested that up to 40% of DCM is inherited, and mutations in over 40 different genes have been implicated in its pathogenesis [4]. For many years now, DCM has been considered to be an irreversible condition, with a late diagnosis and a poor prognosis, but the advances in pharmacological and surgical treatment have significantly improved the prognosis of DCM, with an estimated survival of up to 85% at 10 years free from heart transplantation [5].

However, nowadays, by the time the patients are diagnosed, they often tend to have severe contractile dysfunction and remodeling of both ventricles, reflecting a long period of asymptomatic silent disease progression and the development of myocardial fibrosis. Detailed characterization of these parameters has a pivotal role in the prognostic stratification of DCM patients and in improving clinical management. In this setting, cardiac magnetic resonance (CMR) emerges as a reliable imaging modality providing functional and structural data and fundamental information regarding tissue composition [6]. Using imaging techniques such as late gadolinium enhancement (LGE) and qualitative/quantitative parameters including T1 mapping, T2 mapping, and T2* mapping, tissue characterization is useful for the differential diagnosis of secondary causes of DCM and in the assessment of the probability of ventricular remodeling with a potential role in guiding individualized treatment strategies [7].

The purpose of this article is to review the utility of CMR in the diagnosis and management of idiopathic DCM, providing a deep overview of clinical applicability of standard and emerging CMR contrast-based techniques. This is consistent with current evidence and guidance moving beyond traditional prognostic markers such as ejection fraction.

## 2. Evolving Role of CMR in Cardiomyopathy

Non-ischemic DCM is a range of conditions that primarily affect the heart muscle with a heterogeneous clinical presentation and natural history. Determining the etiology of each type of cardiomyopathy is of major clinical importance as it has implications for optimal treatment strategies and prognosis. Cardiovascular imaging plays an integral part in diagnosis, etiology, risk stratification, and prognosis [1,2,3,4,5,6,7,8]. First, through its ability to characterize the myocardial tissue using multiple different imaging parameters, CMR provides insights into the etiology of underlying heart failure and its prognosis. The latest European Society of Cardiology guidelines (ESC 2023) in cardiomyopathy recognize that CMR should be considered (Class IIa, Level C) in DCM, to distinguish between an ischemic or non-ischemic etiology, and in HCM, for the differential diagnosis, and assessment of the diagnostic criteria [9].

An accurate and reproducible cardiac evaluation always includes chamber size quantification, myocardial wall thicknesses, ventricular function and mass measurement using traditional cine sequences, steady-state free precession (SSFP), in short and long axis (2, 3, and 4 chamber) view and tissue characterization sequences. Late gadolinium enhancement (LGE)-identified fibrosis correlates with histological changes, fibrosis biomarkers and can assess myocardial viability [10]. The pattern of LGE allows for the differential diagnosis between ischemic and non-ischemic DCM with good specificity [11]. Μid-wall fibrosis represents an independent predictor of mortality and morbidity beyond left ventricular ejection fraction (LVEF) in DCM. A cohort study of 427 consecutive patients with DCM implicated the prognostic value of midwall LGE in a comparison between DCM patients with and without LGE which showed that the presence and extent of LGE was associated with increased death probability (26.8% vs. 10.6%) and with an increased risk of arrhythmic event (29.6% vs. 7%) [12].

T1 mapping is a novel and robust CMR technique which offers quantitative measures of the myocardial signal. It creates a pixel-wise parametric map, in which each pixel reflects the absolute value of T1, coded in color [13]. Moreover, it directly measures the extracellular volume (ECV) fraction from T1 values before (native T1) and after administration of gadolinium. According to recent studies, in patients with DCM, ECV and native T1 are emerging as prognostic predictors of mortality independent of the presence of both LVEF and LGE [12]. Furthermore, an increased native T1 value seems to be present as an early imaging marker of adverse outcomes before the presence of LGE [13]. The presence of fibrosis visible microscopically in CMR on LGE images also represents a risk factor for patients with hypertrophic cardiomyopathy (HCM). The presence and extent of fibrosis in LGE correlates with the risk of sudden cardiac death (SCD) [14]. The extent of LGE appears to have more discriminatory value than its presence, in particular when LGE is ≥15% of the left ventricular (LV) mass, which demonstrated a significant increase in SCD risk [15]. Increased native myocardial T1 values and an elevated ECV fraction were found in HCM, even in non-hypertrophic segments with preserved contraction function or in patients without LGE, suggesting that myocardial tissue remodeling may precede morphological and functional changes [16]. Also, in patients with arrhythmogenic cardiomyopathy, the CMR became crucial for diagnosis and risk stratification for arrhythmic events. A study showed that CMR was an independent predictor of ventricular arrhythmias, and regional wall strain assessed using cine CMR reliably predicts arrhythmogenic ventricular tachycardia substrate [17].

## 3. Traditional Risk Stratification Approach in DCM

SCD, secondary to arrhythmia, remains a fatal risk in approximately 30% of those with DCM [18], and an implantable cardiac defibrillator (ICD) is an effective strategy to prevent SCD. Current guidelines recommend selection for ICD based on an ejection fraction (EF) less than 35% [19]; however, most SCD occurs in those with preserved systolic function (EF > 35%) with no prior indication of primary prevention ICD [20]. The DANISH trial suggests that younger patients may have a survival benefit in association with ICD implantation. Subgroup analysis shows that ICDs provided a significant survival benefit in patients under 70 years old, due to a lower risk of non-sudden death; therefore, their measured sudden versus non-sudden death ratio is higher [21].

LVEF value may vary between different imaging modalities, and CMR has emerged as the gold standard technique for LV volume and function assessment, with the added benefit of providing tissue characterization [22]. Independent predictors of all causes of mortality are an indexed left ventricular end-diastolic volume (LVEDVi) on CMR > 120.5 mL/m^2^ and the presence of more than three segments with midwall fibrosis [23]. Juillière et al. identified in right ventricular ejection fraction (RVEF) an independent predictor of all-cause mortality and a modest predictor of hospitalization due to heart failure (HF) in patients with DCM [24] because of direct right ventricular involvement [24,25,26]. Another parameter that could be evaluated by CMR is the left atrial volume index (LAVi), as a sensitive barometer of LV filling pressure and an important predictor of transplant-free survival and HF risk [27].

Considering the aforementioned patient profiles, it is easy to assume that those patients in the most need of a CMR are patients that are currently living with an implantable cardiac device in order to prevent major cardiac events [28]. As many studies have shown [29], scanning the myocardial contractility pace with the device on maintenance generates images mirroring the actual heart function but might be catastrophic for the device or may lead to major arrhythmic events during examination, which puts the patient at enormous risk. On the other hand, switching off the device produces unrealistic images during CMR, mispresenting the myocardial condition, which actually is the case in each patient currently receiving the most appropriate therapy (CRT) [30]. To overcome that obstacle, the advent of devices including the setting ‘’MR safe mode’’ lead us to be capable of scanning while the device is working with no risk to the health of our patient or the device [31]. This is a chance to overcome the justified uncertainty by physicians worldwide while they provide the most appropriate care to patients which is meticulous and realistic imaging.

## 4. LGE as an Emerging Risk Stratification Method in DCM

Risk stratification in patients with DCM defines the risk of ventricular arrhythmias and sudden cardiac death. During the risk assessment, the presence of LGE modifies the prognosis, defining a worse outcome [32]. Becker et al. [33] found an increased risk of adverse cardiovascular events in patients with LGE compared to those without microscopically observed fibrosis. In subjects with DCM, LGE predicted the endpoint of cardiovascular mortality with a pooled OR of 3.40 and ventricular arrhythmic events of 4.52. Alba et al. [34] identified LGE as an adverse prognostic value in a population of 1672 individuals with DCM. The presence of LGE (39%) was associated with an annual risk of SCD or appropriate ICD shock of 4.0%. Instead, Di Marco et al. [35] highlighted the strong adverse predictor of LGE across the entire LVEF. Patients with an LVEF between 21% and 35% and the absence of LGE were at low risk compared to individuals with LGE present and an LVEF > 35%.

LGE is present in one out of three patients with DCM, and the non-ischemic pattern is the most common, with a midwall or subepicardial distribution usually identified (Table 1). Different types of patterns and locations may be identified as additive prognostic markers. Subepicardial distribution, a ring-like pattern, and a septal or multiple-site location are associated with increased adverse arrhythmic risk. In addition, in patients with midwall fibrosis, Assomull et al. [36] found a high incidence of sudden cardiac death and ventricular arrhythmias. Gulati et al. [12] also documented an increased risk of sudden cardiac death in these subjects, independent of the LVEF. Furthermore, several studies documented an increased risk with the coexistence of multiple patterns [34,35]. On the other hand, the site of distribution also modifies the prognosis. Claver et al. [37] described a high incidence of sudden cardiac death in patients with LGE in the septum and free wall compared with those in the septum only. Instead, LGE observed in the right ventricular insertion is considered an unspecific pattern. In a large cohort study of patients with idiopathic DCM, a significant lower incidence of arrhythmic events in subjects with the right ventricular insertion points pattern (IP-LGE pattern) compared with the IP and LV–LGE pattern (LGE present in both right ventricular insertion points and the left ventricle) was documented. In addition, a similar incidence was found with LGE-negative patients. Finally, the ischemic pattern has been also detected in a low number (~5%) in DCM patients [37]. De Angelis et al. [38] identified this LGE pattern with worse long-term outcomes with an adjusted hazard ratio of 2.1. Of note, a recent prospective observational cohort study of 254 patients with early non-ischaemic DCM assessed by CMR by our group (median follow-up 7.9 years) looking into the natural history of fibrosis showed that early DCM is not a benign condition; fibrosis develops early in the phenotypic course and in-depth characterization enhances risk stratification and might aid clinical management [39].

## 5. T1 and ECV Quantification in DCM

T1 mapping and extracellular volume (ECV) may improve the risk stratification in patients with DCM. This non-invasive assessment of myocardial fibrosis could be valuable, especially in those with negative LGE.

Cadour et al. [40] conducted a prospective longitudinal, multicenter study with a 2-year follow-up of 225 patients with a formal diagnosis of DCM. They documented that T1 mapping was an independent predictor of arrhythmia-related events in this population. Also, it was found that the prognostic role of T1 mapping was significantly associated with cardiac death and heart transplantation in patients with both positive and negative LGE [41]. Puntmann et al. [48] followed 637 DCM patients in a prospective longitudinal, observational, multicenter study for a median of 22 months. They highlighted that T1 mapping was an independent predictor of all-cause mortality in DCM. A recent meta-analysis [49] showed the significant prognostic value of T1 mapping in a population of 1242 patients with DCM. Specifically, HR was 1.20 for a composite outcome of mortality and morbidity.

Likewise, ECV appears to be another promising tool, adding value in prognosis stratification in patients with DCM. Rubiś et al. documented that ECV was an independent predictor of ventricular tachycardia in DCM [42,49]. In addition to the occurrence of arrhythmia-related events, it was shown that ECV with a value > 32.1% was an independent predictor and associated with a four-fold increase in the risk of heart failure events [40]. Furthermore, ECV demonstrated a prognostic value even in patients with DCM and negative LGE [41]. Vita et al. [42,50], in 240 individuals with DCM followed for a median of 3.8 years, detected a 2.8-fold adjusted increase risk of major adverse cardiovascular events (MACE) for every 10% increase in ECV (*p* < 0.001). Instead, Kiaos et al. [49] showed the association of ECV with an HR of 1.38 for a composite outcome of mortality and morbidity.

T1 mapping and ECV are promising parameters in the risk stratification of patients with DCM. Larger and multicenter studies are required for widespread clinical use and to standardize the application of these parametric mapping sequences. T1 mapping and ECV could add key prognostic indications in the decision-making algorithm in DCM with negative LGE. Indeed, these techniques are sensitive to assess disease in the initial process and add new prognostic directions of the established clinical and echocardiographic parameters as used until present. A multiparametric approach (LGE, T1 mapping and ECV) may significantly improve arrhythmia risk stratification in DCM patients.

Besides the alteration of myocardium, in patients with DCM, alteration in the haemodynamic can be detected in dilated and dysfunctional ventricles as well [51]. Four-dimensional flow acquired by an MRI scan is a method used to assess the multidimensional blood flow dynamics in the heart and the great vessels in different patient groups with heart conditions [52], aiming to translate those measurements with useful clinical insights. The aforementioned study conducted by Eriksson et al. [51] comparing healthy adults and patients with idiopathic DCM confirmed that the haemodynamic forces acting in both the long and short axis (Lax and Sax) of the heart are different in both early and late diastolic filling of ventricles in patients with DCM and the control group. This study underlined that different haemodynamic forces alter the wall stress that myocardium experiences, hence leading to a heterogenous heart remodeling. In the long run, the set target is to use these findings to prevent or explain heart failure to a patient.

## 6. Advanced LGE-Based Techniques: Gray Zone Fibrosis and Myocardial Entropy

As we delve into cardiac imaging, novel advanced techniques gain ground and may lead to promising results. Myocardial entropy (or left ventricular entropy) is a measurement derived from the intensity of the signal in myocardium during LGE techniques when performing a CMR [44]. As mentioned in the previous study, the aim of its use is more in-depth tissue characterization, provided when LGE images are obtained, by examining the extension of fibrosis in the entire left ventricle beyond the visual and signal intensity thresholds that are currently used in LGE imaging. Many studies in the field have confirmed that entropy is an independent prognostic value for mortality [53] or arrhythmic events [44] in patients presenting with systolic dysfunction or preserved LVEF. The previously mentioned study also clarifies that entropy can also be measured in the absence of macroscopic scar in LGE images with minimal postprocessing, designating concealed conditions of cardiac risk, which can usually be the case for these specific patients.

**Figure 1:**
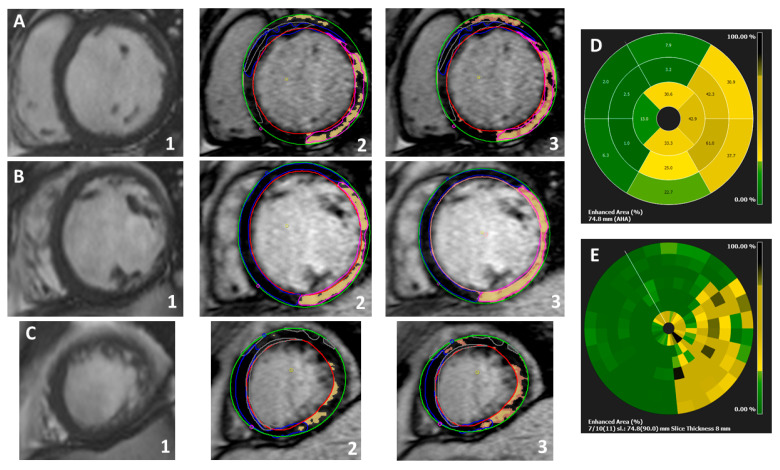
Extensive inferior and lateral near-transmural replacement fibrosis, alongside patch of subepicardial fibrosis in the basal anterior segment in a non-ischaemic familial cardiomyopathy case (Filamin-C positive); example of myocardial and gray zone fibrosis analysis using Circle42©; (**A**) steady-state free precession (SSFP) cine at the basal segment (**1**), LGE quantification (full-width half maximum technique-FWHM) (**2**), and gray zone analysis (orange pixels surrounding the ‘core’ fibrosis represented by the ‘yellow’ pixels) (**3**). (**B**) Cine SSFP at the mid segment (**1**), LGE quantification (FWHM) (**2**) and gray zone analysis (**3**). (**C**) Cine SSFP at the apical segment (**1**), LGE quantification (FWHM) (**2**) and gray zone analysis (**3**). (**D**) Auto-generated graph depicting the enhanced area (%) according to AHA segmentation. (**E**) Auto-generated graph representing the enhanced area (%) using multiple segments analysis (slice thickness 8 mm).

Gray zone fibrosis (GZF), represents an admixture of viable and non-viable myocardium and has emerged as a marker of the arrhythmogenic substrate and has been linked to arrhythmic events in numerous studies [43] (Figure 1). The concept behind its use is based on the pathophysiological role of fibrosis in arrhythmogenic events, as well-functioning myocardium is being interrupted by fibrotic corridors, resulting in a blockage in electrical conduction and activating the mechanism of re-entry ventricular arrythmia which can lead to fatal results for the individual. Thus, this study concluded that patients at the lowest tertile of GZF had a low risk of ventricular arrythmias or cardiac death, whereas those in the highest tertile had a high risk of ventricular arrythmias (VA) or SCD [43]. The threshold and different methods of GZF are modified and applied to each investigator’s preferences; therefore, a more robust frame of values should be established. Also, this study underlines that the optimum cutoff value of GZF will also depend on the study population as well as the nature of the cardiomyopathy underneath. Nevertheless, the value of the method cannot be underestimated by those limitations, as it has been proven to provide realistic risk stratification and give prominence to the patients in actual need of ICD implantation in contrast with the traditional criteria used, such as LVEF [43].

## 7. Limitations of CMR in DCM

CMR is the gold standard method when assessing cardiomyopathies; however, we must acknowledge some limitations. Certainly there are known challenging studies, for instance, in patients with poor respiratory cooperation or arrhythmias, which may render even volumetric analysis suboptimal. We have increasingly appreciated different patterns linked to certain high-risk genotypes such as Filamin C or Desmoplakin. As mentioned before, LGE patterns can be related to certain DCM types and are predictors of all-cause mortality, cardiovascular hospitalization, SCD, and ventricular tachycardia [54]. However, LGE patterns are present in approximately 60–90% of DCM cases which reflects a great heterogeneity and thus a limited specificity in some cases [55]. Moreover, we are far from understanding all of the different genotypes in inherited DCM, their pathophysiology and their phenotypic expression. Also, the role of native mapping has been explored and is currently being further evaluated in cardiomyopathy; however, there are no validated reference numbers in patients with implantable devices.

## 8. Future Directions

Myocardial strain describes myocardial deformation and is a parameter of myocardial function in addition to EF. The two main techniques in the assessment of strain via CMR are MR tagging and MR feature tracking (MR-FT). CMR-FT is currently a method with great potential, as tracking can be applied to standard cine images, and no additional sequences are needed. Cut-offs for strain values vary among methods, modalities, and software [45]. Recent studies have investigated the association between myocardial fibrosis and strain abnormalities. Similar to LGE and mapping, abnormal strain values appear to be associated with prognosis in DCM patients. A study using CMR-FT found impaired global longitudinal strain (GLS) and mean longitudinal strain to be an independent prognostic parameter for a composite cardiac endpoint of cardiac death, heart transplantation, and aborted SCD [56]. Another multicenter study showed that CMR-FT-derived GLS is a powerful independent predictor of mortality, incremental to common clinical and CMR risk factors, including ejection fraction and late gadolinium enhancement [57]. On the other hand, Pi SH et al. found no prognostic value in the LV strain in a DCM high-risk population [47]. Overall, the results on strain in the risk assessment are promising.

Another promising novel tool is the use of machine learning (ML). A study developed an ML model that included systolic blood pressure, left ventricular end-systolic and end-diastolic volume indices, and late gadolinium enhancement (LGE) extents on CMR imaging. The model showed excellent performance in predicting adverse events in DCM patients with severely reduced LVEF [46]. Further studies are needed to validate these new techniques and to include them in clinical practice.

## 9. Conclusions

From an imaging perspective, CMR is currently the reference method for the study of dilated cardiomyopathy. With the ability to analyze myocardial tissue in addition to the calculation of volumes and systolic function, it allows discrimination between different etiologies and enables more precise risk assessment. Many studies have been performed and many more are currently in progress regarding the use of CMR novel tools in risk stratification for patients with DCM.

## Figures and Tables

**Table 1:** Summary of classic and novel techniques used in non-ischaemic DCM.

**Technique**	**Description**	**Current Use**	**Clinical Applications**
**A. Traditional techniques**			
1. Echocardiogram [19] Priori, S.G. et.al	EF measured by U/S (2D)	To determine if the patient is eligible for ICD implantation for prevention of SCD	NYHA status is taken into consideration, but overall inadequate risk stratification method
2. Non contrast CMR [23] Guaricci, A.I., et al.	Gold standard technique for LV volume and function assessment providing tissue characterization	Assessment of fibrosis and its location gives predisposition for symptoms or events may be observed	Calculates RVEF, LVEDi which are useful mortality predictors. LAVi is an important predictor of transplant-free survival and HF risk.
**B. Recent and advanced imaging techniques**			
1. LGE-based fibrosis [32] Hammersley et al. [36] Assomull, et al.	Gadolinium-based myocardial fibrosis.	It is present in 1/3 patients with DCM, and the non-ischemic pattern is the most common with a midwall or subepicardial distribution usually identified	Increased risk of adverse cardiovascular events when detected. Strong predictor of cardiovascular mortality across the entire LVEF. Site of distribution modifies the prognosis.
2. Non-contrast (native) T1 mapping [40] F. Cadour et al. [41] S. Li et al.	Non-contrast parametric mapping to assess myocardial microstructure based on T1 tissue properties	Method used increasingly in patients with cardiomyopathy	Independent predictor of arrhythmogenic events in DCM patients [40]. Associated with cardiac death and heart transplantation in patients with both positive and negative LGE test [41].
3. ECV Quantification [40] F. Cadour et al. [42] P. P. Rubiś et al.	Parametric mapping pre-(native) and post contrast administration	A method for the evaluation of focal and diffuse myocardial fibrosis	Independent predictor of ventricular tachycardia in DCM patients. Independent predictor and associated with a four-fold increase in risk of heart failure events. Improves risk stratification in DCM patients, due to a more advanced characterizing process particularly when LGE test is negative.
4. Gray Zone Fibrosis [43] Leyva F., et al.	Advanced LGE quantification method	An admixture of fibrosis and viable tissue thought to be a substrate for ventricular arrhythmias	Role in prediction of life-threatening arrhythmias and prognosis.
5. Myocardial Entropy [44] P. Antiochos et al.	Measurement derived from Shannon’s entropy mathematical models	Advanced texture analysis of fibrosis	Role in prediction of life-threatening arrhythmias and prognosis.
6. MR feature tracking (MR-FT) [45] M. S. Amzulescu et al.	Myocardial deformation in addition to EF traditionally measured	CMR-FT is a method with great potential, as tracking can be applied to standard cine images, and no additional sequences are needed.	GLS and mean longitudinal strain impaired—may be an independent prognostic parameter.
**C. Novel Imaging Assessment**			
7. Machine Learning Techniques- (ML) [46] S. Shu et al.	The understudied ML model included systolic blood pressure, left ventricular end-systolic, end-diastolic volume indices and late gadolinium enhancement (LGE) extents on CMR imaging.	Most ML techniques are not yet established due to limited amount of clinical studies performed.	The particular model showed excellent performance in predicting adverse events in DCM patients with severely reduced LVEF [47].

Abbreviations: CMR: cardiac magnetic resonance, DCM: dilated cardiomyopathy, ECV: extracellular volume, EF: ejection fraction, LGE: late gadolinium enhancement, LV: left ventricle, LVEF: left ventricular ejection fraction, SCD: sudden cardiac death, ICD: implantable cardioverter defibrillator, LVEDVi: indexed left ventricular end-diastolic volume, LAVi: left atrium volume index.

## Data Availability

The original contributions presented in the study are included in the article, further inquiries can be directed to the corresponding author/s.
